# Chest wall and diaphragm reconstruction; a technique not well established in literature – case report

**DOI:** 10.1186/s13019-021-01577-w

**Published:** 2021-07-09

**Authors:** Riad Abdel Jalil, Mohamad K. Abou Chaar, Obada Al-Qudah, Hanna Kakish, Salam Elfar

**Affiliations:** 1 Department of Thoracic Oncology, King Hussein Cancer Center, Queen Rania Al Abdullah Street, P.O. Box 1269, Amman, 11941 Jordan; 2 Department of Surgery, King Hussein Cancer Center, Amman, Jordan; 3 Department of Research, King Hussein Cancer Center, Amman, Jordan; 4 Department of Pharmacy, King Hussein Cancer Center, Amman, Jordan

**Keywords:** Chest wall tumor, Chest wall reconstruction, ePTFE mesh, Case report, Surgical technique

## Abstract

**Introduction:**

Regardless of its rarity, and indolent clinical course, chest wall tumor places high morbidity and burden on patients especially when invasion to a neighboring structure is found. Once detected, surgery is the cornerstone for treatment of such etiology combined with chemo-radiotherapy. In order to maintain intact respiratory function, chest wall reconstruction must be performed whenever resection is done. Herein, we present a case of chest wall tumor that necessitated three ribs and part of hemidiaphragm resection and reconstruction with optimal post-operative results.

**Case presentation:**

A 27-year-old male patient who had chest wall and diaphragm reconstruction for a chest wall Ewing sarcoma, using a single patch of expanded polytetrafluoroethylene (ePTFE) mesh with diaphragm implanted into the middle of the mesh. There were no immediate nor post-operative complications. The patient received post-operative radiotherapy with good functional and cosmetic results.

**Conclusion:**

We present a novel and safe technique for combined chest wall and diaphragmatic resection following excision of an invading tumor while ensuring cosmesis and functionality of the ribcage as well as the diaphragm.

## Introduction

Chest wall tumor (CWT) is a generic term that includes primary neoplasm, both benign and malignant, arising from cartilage, bone, muscle, fascia, adipose tissue, skin, and vessels of the chest wall. Secondary neoplasm includes invading breast or lung malignancies, and distant metastasis [1]. Primary tumors of the chest wall are rare heterogeneous group of neoplasms with an incidence less than 2% [2] with a high rate of malignancy reaching up to 80%, almost half of which arise from the bone and cartilage of that confined space [3]. Once suspected, computed tomography (CT) scan and magnetic resonance imaging (MRI) studies must be carried out for better understanding of the tumor’s etiology, followed by an adequate biopsy, all of which must be guided via a multidisciplinary approach for treatment planning [1, 4, 5]. Till this day, surgical resection followed by reconstruction is the corner stone for management of such cases in combination with chemo-radiotherapy whenever necessary [6].

Here, we describe a case of Ewing sarcoma located in the inferior border of the right chest wall with invasion of the lateral abdominal wall muscles as well as invading the diaphragm. An expanded polytetrafluoroethylene (ePTFE) mesh has been used for reconstructing the post-excision defect.

## Case presentation

**Fig. 1:**
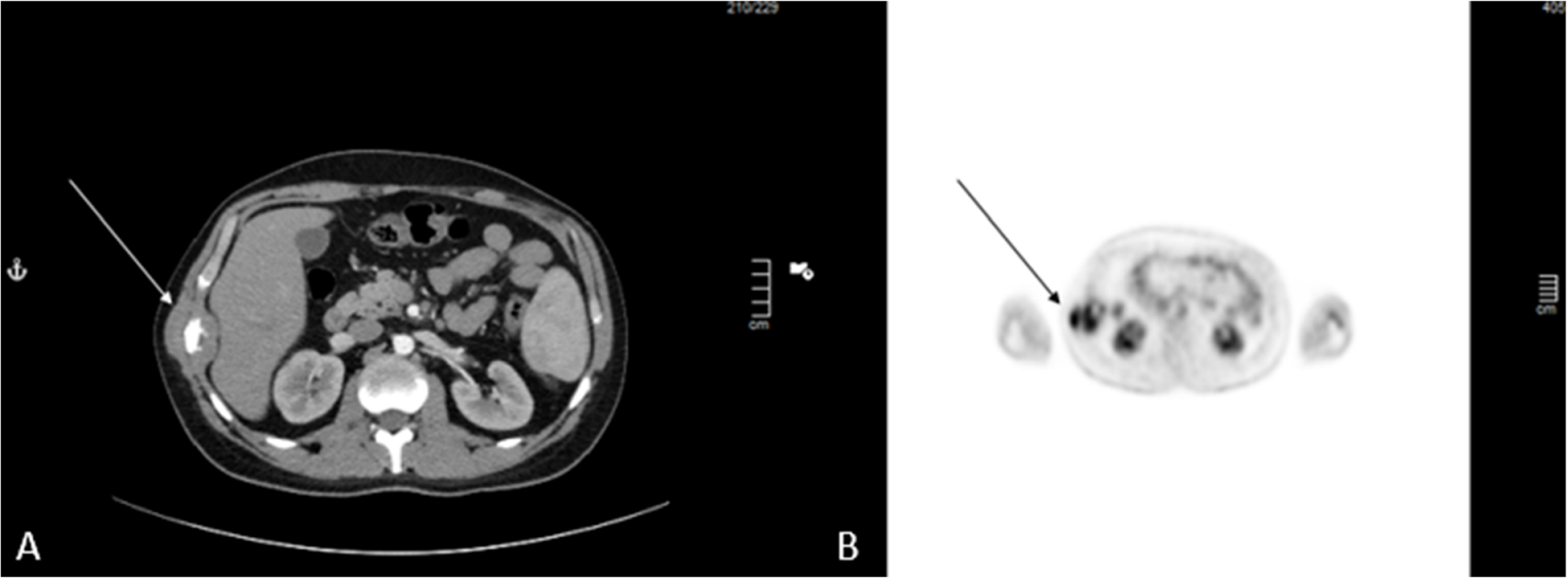
Preoperative CT and PET scans showing tumor location

**Fig. 2:**
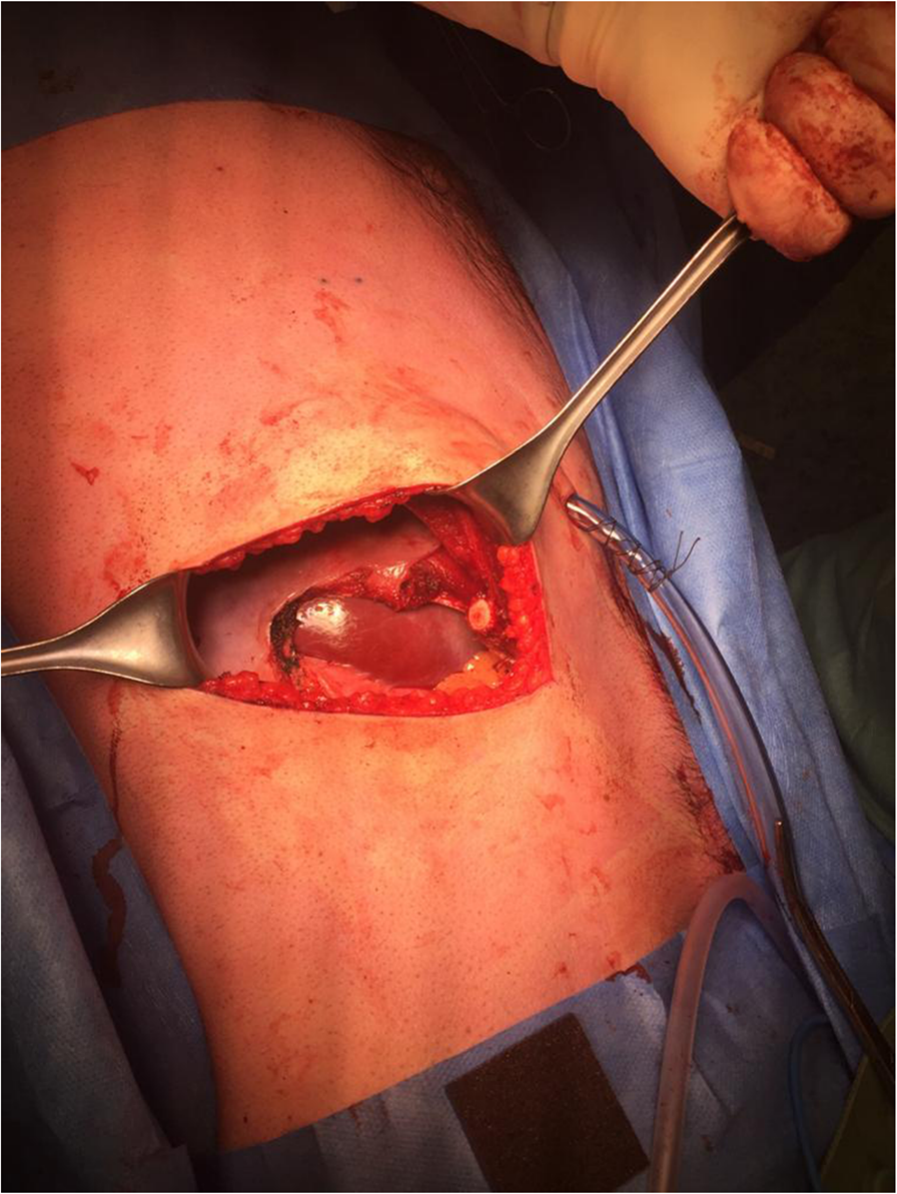
Intraoperative image showing complete resection of the mass with invaded surrounding structures

**Fig. 3:**
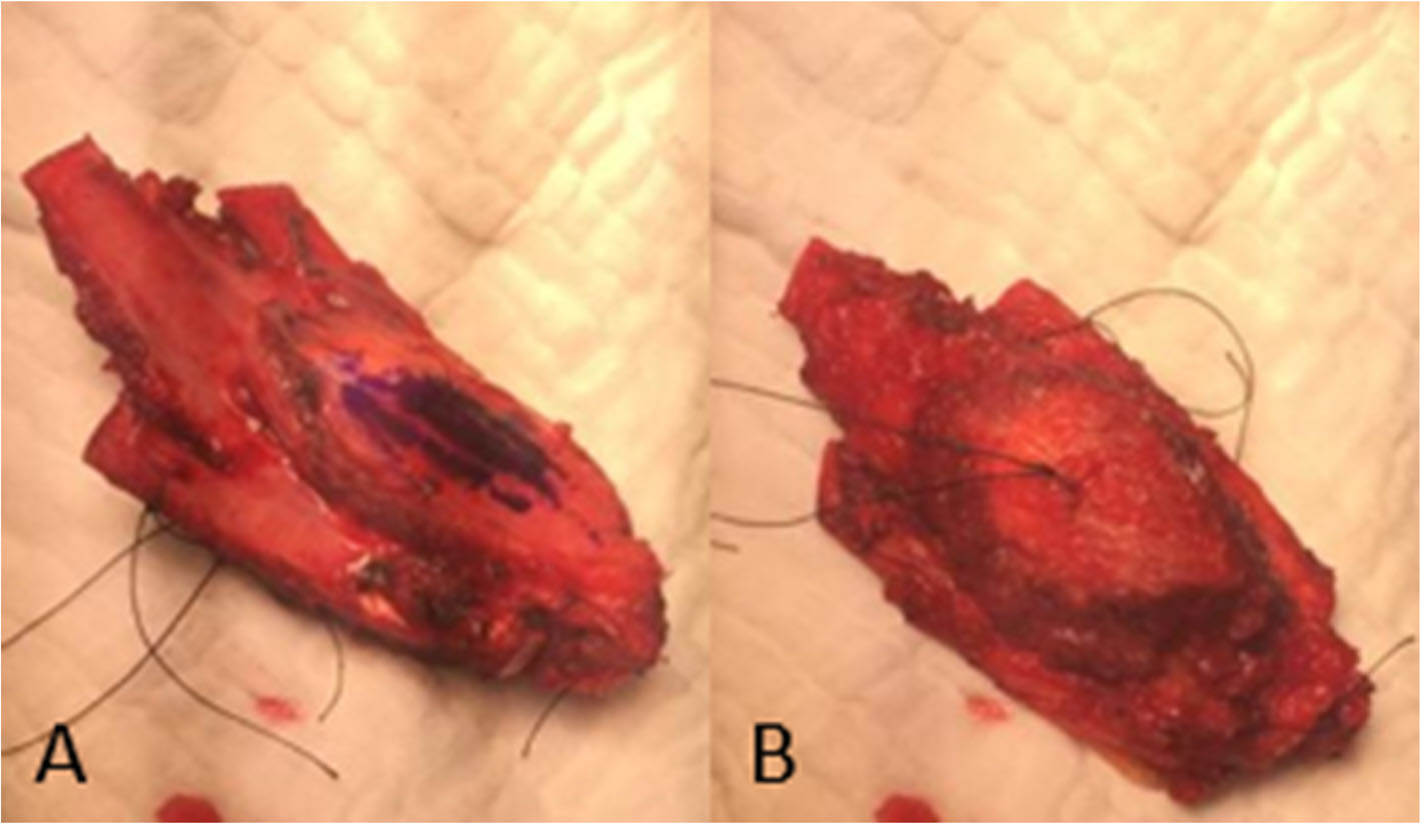
The tumor after complete excision

**Fig. 4:**
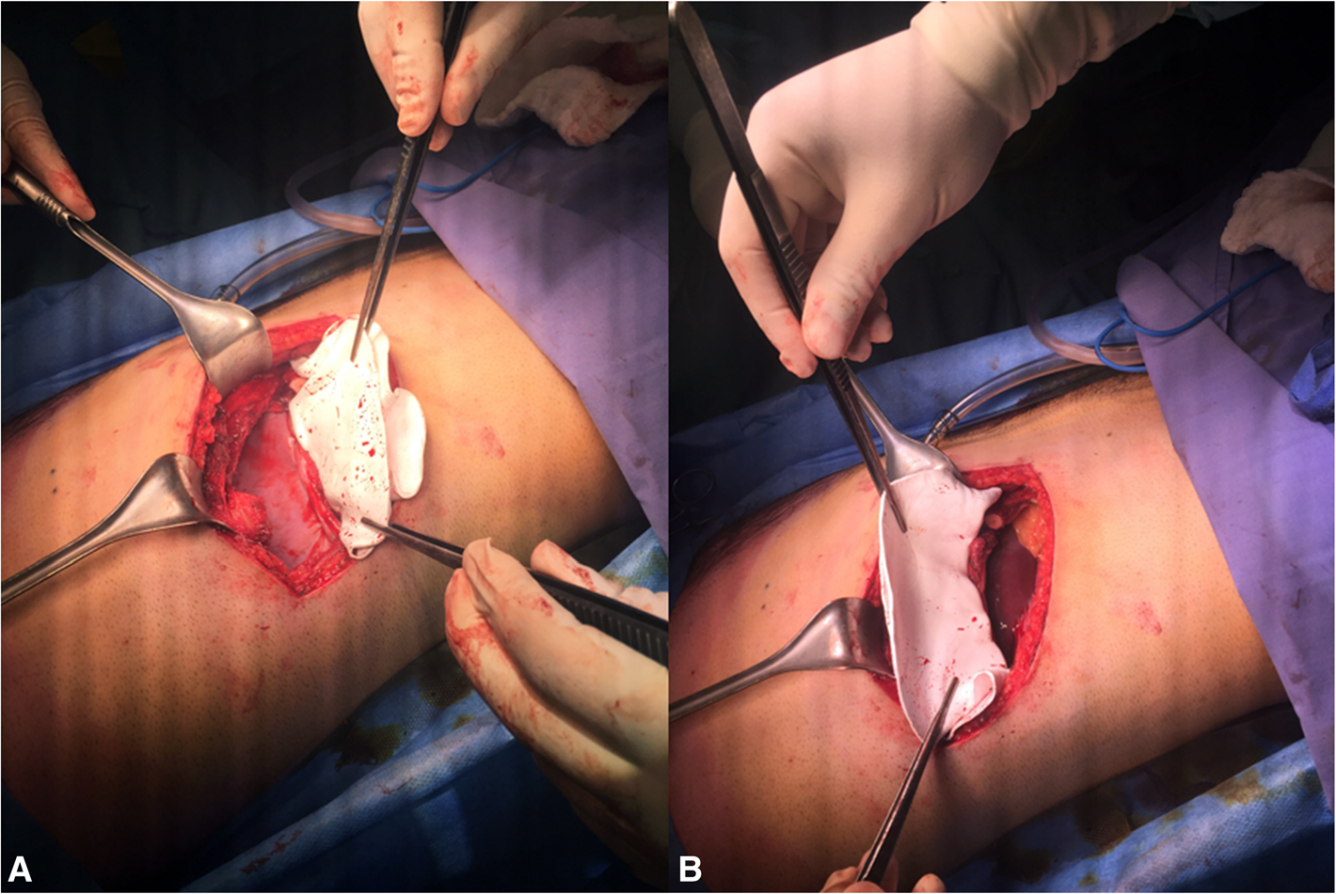
Intraoperative image showing how diaphragm reconstruction

**Fig. 5:**
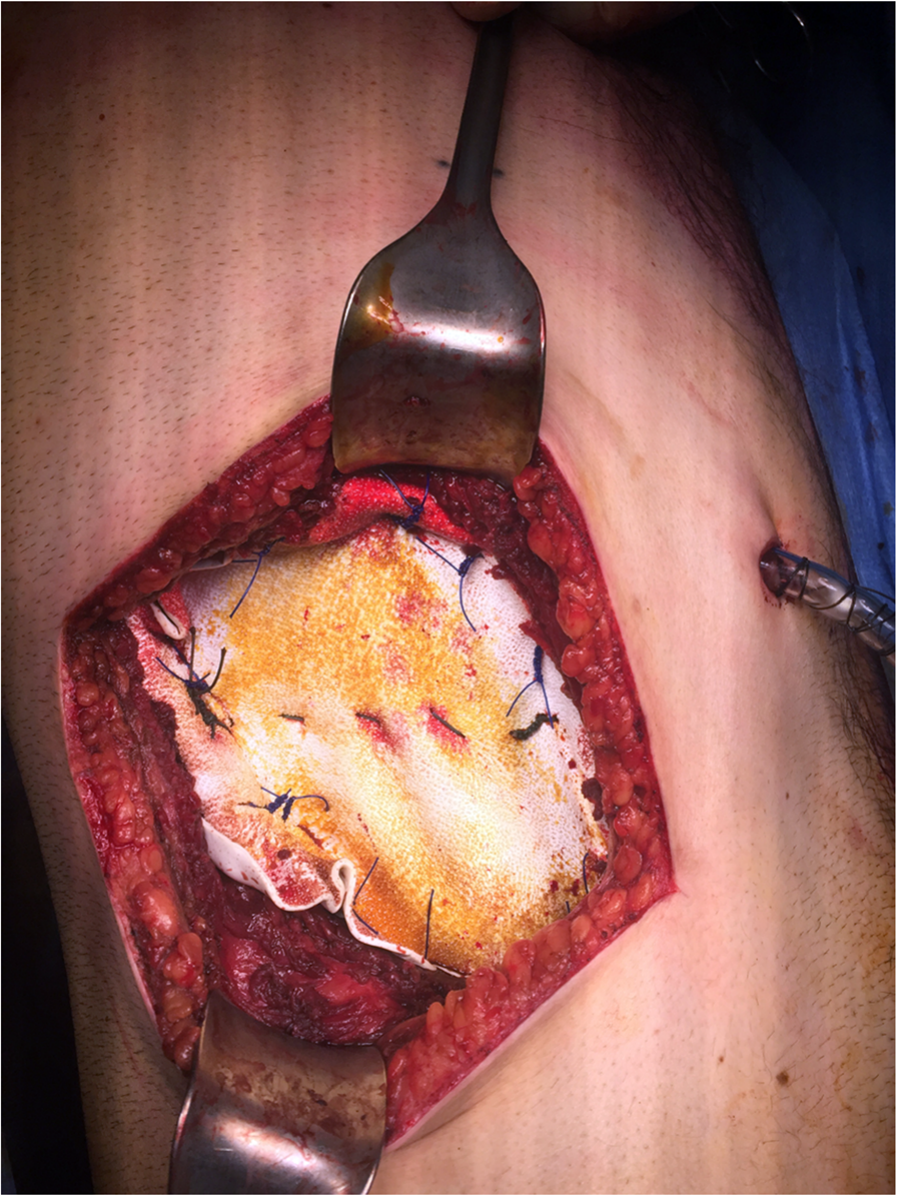
Intraoperative image showing complete reconstruction of the chest wall defect

A 26-year-old male patient, not known to have any comorbidities, has a negative family history of malignancy, non-smoker, and with a history of left chest wall (7th and 8th ribs) Ewing sarcoma treated with surgical resection followed by 14 cycles of alternating Vincristine (1.5 mg/m^2^/dose) – Doxorubicin (37.5 mg/m^2^/day) – Cyclophosphamide (1200 mg/m^2^) – MESNA 360 mg/m^2^ and Ifosfamide (1800 mg/m2/day) – Etoposide (100 mg/m^2^/day) – MESNA (360 mg/m^2^) (VDC/IE Protocol) and 28 fractions of radiotherapy finished in 2008 and was declared on remission and kept on regular follow-up. On January, 2019 he presented with a right sided muscular chest pain of one-month duration associated with a non-tender mass that started to grow gradually. His initial laboratory results showed a hemoglobin 16.5 g/dl, white blood cell count of 5.8 10^3^/μ, platelet count of 235 10^3^/μ, and normal values for liver and kidney function tests. On physical examination, a right sided chest wall mass located close to the anterior axillary line, measuring about 5 cm in diameter. The mass was firm, non-tender, fixated to the chest wall, and without overlying skin changes. A computed tomography (CT) scan of the chest with contrast showed right chest wall mass involving the right tenth rib laterally with large soft tissue component indenting the liver surface. A positron electron tomography/CT (PET/CT) scan revealed a hypermetabolic mixed lytic/sclerotic bony lesion within the right 10th rib laterally associated with large heterogeneous soft tissue mass extending externally to involve lateral abdominal wall muscles and medially pushing the peritoneum medially without involvement of abdominal organs. This soft tissue mass measuring about 6 cm in maximum diameter with SUV MAX = 5 (Fig. 1). Ultrasound guided mass biopsy was done and showed tumor cells positive for cluster of differentiation 99 and friend leukemia integration-1 suggestive of recurrent primitive neuroectodermal tumor (PNET)/Ewing sarcoma. The patient and his family were consulted and a multidisciplinary clinic meeting decided to proceed with three cycles of Irinotecan 36 mg that resulted in mild regression of soft tissue component of the bony lesion from 5.5 cm to 3.5 cm. he was then scheduled for surgical excision. Under general anesthesia and in a left lateral position, an oblique skin incision was done over the level of the right 10th rib. After creating a superior and inferior subcutaneous flaps, the mass was identified in the 10th rib. A wide local resection of the mass including the involved rib, with a 4 cm safety margins anteriorly and posteriorly, in addition to part of the 9th and 11th ribs, and part of the diaphragm and abdominal wall resulting in a 15 X 8 cm chest wall defect (Figs. 2 and 3). We used a single patch of ePTFE mesh (Bard Dulex Mesh, Davol Inc., USA) for chest wall reconstruction, measuring 15 cm X 19 cm X 2 mm. After placing the mesh into a satisfactory position, a diaphragm reconstruction was done by suturing it directly along the middle part of the interior surface of the mesh using continuous runs of Ethibond Excel Polyester Suture (Ethicon, USA) (Fig. 4). The edges of the mesh were then secured with interrupted runs of Prolene size 0 suture to the 8th and 12th ribs (Fig. 5). Primary closure of the latissimus dorsi muscle over the mesh was done, then suturing of the subcutaneous tissue and skin. The operation had 200 ml of blood loss, did not require any blood transfusion and had a smooth course. The patient was kept in the intermediate care unit (IMU) for 24 h post-operatively and then chest tube was removed on post-operative day one with an output of 20 ml and the abdominal drain was removed on the day after with a total output of 340 ml of serosanguinous fluid. The patient was discharged on the third post-operative day with a smooth hospital-stay course. Pathology of the specimen confirmed a diagnosis of PNET/Ewing sarcoma with negative resection margins. Patient then received adjuvant chemotherapy (2 cycles of IE and 2 cycles of VDC) and radiotherapy (50.4Gy/28 fractions over II phases). Unfortunately, a year after the procedure he was found to have multiple metastatic lesions involving left subpleural space, bilateral lungs, and bone. He is currently on Cyclophosphaide and Topotecan and with good partial response and intact pulmonary function 2-years post-operatively.

## Discussion

All CWTs should be considered malignant until proven otherwise. Wide resection with the achievement of microscopically negative margins should be the goal of surgical resection to improve local control and to prevent local recurrence in both benign and malignant lesions [7].

Wide radical surgical resection is the treatment of choice for malignant chest wall tumors, and is exactly as the name implies, that is; no compromise should be made to reduce the size of the defect [8]. Chest wall resection involves resection of the ribs, sternum, costal cartilages and the accompanying soft tissues. The reconstruction of the defect depends on the site and extent of the resected structure [9]. If the resection is only limited to one rib, an approximation of the overlying muscles without any prosthetic implant is recommended. It was noted that even if a defect is larger than two ribs but located posteriorly in the chest wall, the scapula can be utilized to cover such defect without the need of prosthesis too [10]. Large tumors engulfing more than two ribs necessitate the use of a polyethylene or expanded polytetrafluoroethylene (ePTFE) mesh to cover post-resection defect [11]. Taniguchi et al. reported successful use of polyglycolic acid mesh with cross string sutures and autologous ribs graft in repairing chest wall defect following CWT resection in 9 patients [12]. Myocutaneous flap or omental flap is usually used to cover prosthetic mesh surface in order to avoid internal organ damage and ensure mesh stability [13]. Ge et al. reported the use of a human acellular dermal matrix (HADM) for 7 patients who had CWT and underwent resection. After implantation, HADM gets infiltrated by host cells, lowering antigenicity and rejection rate. Regardless of its efficacy, its expensive cost makes its use only possible in selected patients [14].

Diaphragm involvement by inferiorly located CWT, which is mostly due to invasion rather than primary neoplasm of the muscle itself [15], entails diaphragm as well as chest wall reconstruction. Such procedure entails meticulous planning and surgical technique to ensure adequate chest and diaphragm physiologic function. Kuwahara et al. concluded that either primary diaphragm suture or mesh coverage, in association with chest wall defect resection and mesh reconstruction, can achieve water-tight and air-tight seal of the chest cavity without paradoxical chest movement or increase in mortality. It was also recommended not to perform primary closure of the diaphragm if the distance between the edge of the remaining part of the diaphragm and chest wall is more than 3–4 cm, in order to avoid a flat drum-head diaphragm with loss of its function [16]. Altunkaya et al. reported a case of a huge chest wall mass engulfing the 8th and 9th ribs with diaphragmatic invasion. Post-resection, a 15X10 cm diaphragm defect was covered using PTFE mesh, and the 20X15 cm chest wall defect was covered by Mersilene mesh and methylmetacrylate sandwich graft [17]. Rathinam et al. implanted a custom-made inverted Y polyethylene methylmethacrylate sandwich flap for 6 patients with diaphragm-invasive CWTs, all of which had excellent functional and cosmetic outcomes [18]. In the case presented, ePTFE mesh provided the needed strength and impermeability for chest wall reconstruction, then the diaphragm was pulled laterally and sutured to the middle part of the inner surface of the mesh. If the diaphragm was sutured to the 8th rib or above, a dysfunctionality would have been observed in the picture of tight flat diaphragmatic surface, and if suturing was performed on the 12th rib, which does not have any attachment to the sternum, an unstable diaphragm that is unable to maintain adequate breathing movements will be encountered. Hence, the diaphragm was sutured to the middle part of the ePTFE mesh to ensure stable ribcage and efficient respiratory function.

## Conclusion

A variety of reconstruction methods have been proposed and attempted for chest wall reconstruction. If diaphragm resection was done, either separate synthetic materials were used to cover each defect separately or a custom-made mesh was utilized for combined reconstruction. We describe a method for reconstructing the chest wall and diaphragm using a single patch of ePTFE without sacrificing functionality of either. Even though the technique proposed is simple, meticulous care must be given to ensure suturing of the diaphragm into the correct position to avoid flattening of its dome shaped surface and rigorous attention should be given to secure air-seal and water-seal to the chest wall in order to preserve the natural breathing mechanism of this cavity.

## Data Availability

NA

## Abbreviations

ePTFE: Expanded polytetrafluoroethylene
CWT: Chest wall tumor
CT: Computed tomography
MRI: Magnetic resonance imaging
VDC/IE: Vincristine-Doxorubicin-Cyclophosphamide/ Ifosfamide-Etoposide
PET/CT: Positron electron tomography computed tomography
PNET: Primitive Neuroectodermal tumor
HADM: Human acellular dermal matrix
